# Influence of Organically-Modified Montmorillonite and Synthesized Layered Silica Nanoparticles on the Properties of Polypropylene and Polyamide-6 Nanocomposites

**DOI:** 10.3390/polym8110386

**Published:** 2016-10-31

**Authors:** Moisés Gómez, Humberto Palza, Raúl Quijada

**Affiliations:** Departamento de Ingeniería Química y Biotecnología, FCFM—Universidad de Chile, Beauchef 851, Santiago 8320198, Chile; hpalza@ing.uchile.cl (H.P.); raquijad@ing.uchile.cl (R.Q.)

**Keywords:** layered silica nanoparticles, nanoclays, polypropylene nanocomposites, polyamide-6 nanocomposites, permeability, mechanical properties, thermal stability

## Abstract

Nanocomposites of layered silica nanoparticles (LSN) obtained by the sol–gel method, and commercial montmorillonite clay Cloisite^®^20A with polypropylene (PP) and Cloisite^®^30B with polyamide-6 (PA6) were prepared by melt blending in order to study their effects on barrier, mechanical properties, and thermal stability. Transmission electron microscopy (TEM) showed that all of the nanocomposites present agglomerated nanoparticles with some degree of individual particles. In barrier properties, LSN dramatically increased the oxygen and water vapor permeability of PP at low loadings (<5 wt %) due to the percolation effect. However, in PP and PA6 nanocomposites with clays, the permeability showed increases and decreases depending on the solubility of the permeating gases with the clays and the polymers. Tensile stress-strain tests otherwise showed that the nanocomposites with clays present an enhancement in the elastic modulus. Meanwhile, with the LSN, a decrease was found due to the formation of agglomerations and voids. Finally, thermogravimetric analysis under inert conditions showed the nanoparticles do not have a significant effect on the thermal stability of the nanocomposites. These results expose the relevance of the type of layered nanoparticle and polymer matrix on the barrier, mechanical, and thermal behaviors of the resulting nanocomposites.

## 1. Introduction

Polymer composite is a multiphase material consisting of a continuous polymeric phase and a dispersed phase, generally an inorganic material. When the inorganic phases in these composites become nanosized, they are called nanocomposites [1,2]. The high level of interest in these materials is based on the possibility of greatly enhancing the properties of a given polymeric matrix, as they combine the advantages of the inorganic material (e.g., rigidity, thermal stability, barrier properties, etc.) and the organic phase (e.g., flexibility, ductility, processability, etc.) [1,2,3]. Moreover, low loadings of nanometric fillers (<10 wt %) in polymeric matrices can significantly improve the performance of these materials, such as barrier, mechanical properties, crystallinity, and thermal stability, amongst others [1,2,3,4].

Polypropylene (PP), a non-polar polymer, is of particular interest due to the fact that it is a versatile thermoplastic material compatible with many processing techniques and is used in many different commercial applications. Among all thermoplastics, PP presents the fastest growing in the recent decades [4,5]. On the other hand, nylon-6 or polyamide-6 (PA6), a polar polymer, is a semicrystalline thermoplastic that has a wide range of engineering applications for its unique combination of good processability, higher mechanical properties and chemical resistance [6,7,8]. Since the pioneering work of Toyota’s research team in the early 1990s, who prepared a reinforced polymer nanocomposite of nylon-6 with montmorillonite [6,9] where increases by a factor of two were obtained in the tensile modulus and strength with 5 wt % of clays [9], extensive research from both academic and industrial groups have followed.

Montmorillonite (MMT) is the most frequently used clay in the preparation of polymer composites [10]. The considerable attention received by this filler derives from both its potential to be exfoliated in the polymer matrix and its structure exhibiting the required stiffness, strength, and dimensional stability; moreover, its low cost, easy access, and layered structure are additional advantages [5,7,10]. When these clays are used as filler in polymers, they are usually chemically modified to allow the exfoliation of their lamellar structures to improve the polymer–particle interaction [7]. One of the most used clays for non-polar polymers, such as PP, is the commercial Cloisite^®^20A (C20A), a montmorillonite, which has been ion-exchanged with dimethyl di(hydrogenated tallow) ammonium chloride [1,5,7]. For polar polymers, such as PA6, Cloisite^®^30B (C30B) is currently used, which is a clay modified by bis-2-hydroxyethyl ammonium chloride surfactant [7,11]. Due to the nanoclay platelet-like structure presenting a high aspect ratio and propensity to accelerate polymer crystallization, nanocomposites with these clays show enhanced mechanical properties, improved solvent resistance, high thermal stability, and, usually, reduced permeability [5,7,10,12]. In PP nanocomposites with 2.5 wt % and 5 wt % of C20A, an increase of 33% and 60% in Young’s modulus, respectively, was found. In thermal properties, the degradation temperature presented an increase of 35 °C with 5 wt % of this clay [4,7]. On the other hand, an increase up to 68% in the Young’s modulus with 5 wt % of C30B in PA6 nanocomposites has been reported. Meanwhile, the degradation temperature showed an increase of 10 °C with 2.5 wt % of this clay [13].

Although PP/MMT and PA6/MMT nanocomposites have presented significant enhancements in their properties, natural impurities remaining in these clays may affect color, magnetic, optical, and mechanical properties, among others [14]. A novel route to solve this problem is to use synthetic silica nanoparticles as fillers by the sol–gel process [3,4]. Two of the major advantages of this method are the mild reaction conditions, such as relatively low temperature and pressure, and the synthesis of highly pure materials [4,15]. Additionally, this method can be adapted in order to produce nanoparticles in a wide range of sizes and geometries [14,16]. The spherical geometry has been studied mostly where mechanical and thermal properties have shown an important enhancement in PP and PA6 nanocomposites. In PP nanocomposites with layered silica nanoparticles, an increase in the elastic modulus of 8% has been reported at 1 wt %, decreasing linearly at higher loadings (≥3 wt %), due to a weak interaction between the polymer and the nanoparticles [15]. In the case of PA6 with spherical silica nanoparticles, increases up to 75% at 5 wt % of them has been found [1]. These improvements can be attributed to the high rigidity of the nanoparticles along with the high interfacial interaction polymer-particles. With respect to thermal properties, on the other hand, the incorporation of nanometer-sized inorganic particles into the polymer matrices typically enhance the thermal stability by acting as a superior insulator and mass transport barrier to the volatile products generated during decomposition [1,4,15,17].

Regarding the permeability of nanocomposites, when layered synthetic silica nanoparticles are used, a decrease in O_2_, N_2_, CO_2_, and other gases was reported in different polymer nanocomposites (PP, PA6, PE, etc.) [18,19]. However, other researchers have found an increase of O_2_, N_2_, and H_2_O permeability in PP/spherical silica nanocomposites [3,4]. In the clay’s cases, increases and decreases in the permeability of O_2_, N_2_, CO_2_, He, and H_2_ gases were also found in PP/MMT [4,7] and PA6/MMT [11,13] nanocomposites. These contradictory results emerge from the different phenomena occurring in the nanocomposites associated with diffusion processes. It is usually widely accepted that the decrease in permeability is mainly due to a longer diffusion path of the permeating gas molecules as a result of an increase in the tortuosity, frequently observed in exfoliated nanocomposites [5,7,8]. The improvement in permeability, otherwise, is typically due to an increase of the polymer free volume in the polymer–particle interface caused by a weak interaction [3,13,20]. This indicates the permeability’s mechanism in polymer nanocomposites is not fully understood yet. In respect to the layered silica nanoparticles, they have not been widely studied and these results, as compared with organically-modified layered MMT, could provide important information for a better understanding of the effect of layered nanoparticles in nanocomposites.

In this article, PP and PA6 nanocomposites were prepared with organically-modified montmorillonites (Cloisite^®^20A and Cloisite^®^30B) and layered silica nanoparticles were synthesized by the sol–gel method in order to contribute to the understanding of the nature of the effect between polymer and layered fillers on the permeability (oxygen and water vapor), mechanical properties, and thermal stability.

## 2. Experimental

### 2.1. Materials

Polypropylene, with a melt flow index (MFI) of 7.5 g/10 min (PP-H401) was obtained from Braskem (São Paulo, SP, Brazil) and used as received. Polyamide-6, with a MFI of 5.9 g/10 min (ultramid B33L) was obtained from BASF (Ludwigshafen am Rhein, Germany) and used as received. Tetraethoxysilane (≥98%, TEOS) and hexadecyltrimethoxysilane (≥85%, HDTMOS) were obtained from Sigma-Aldrich (San Luis, MO, USA). Ammonia (25 wt %) and hydrochloric acid (32 wt % HCl) were obtained from Equilab (Equilab, Madrid, Spain). Commercial clays Cloisite^®^20A and Cloisite^®^30B were provided by Southern Clay Products Inc (Gonzales, TX, USA) and used as received. The antioxidant used in the preparation was a 2:1 mixture of Irganox 1010 and Irgafos 168, respectively. Layered silica nanoparticles were produced using a sol–gel method based on the work of Chastek, et al. [14].

### 2.2. Composite Blending

PP/layered silica, PP/C20A and PA6/C30B were produced by melt-mixing in a Brabender Plasti-Corder (Brabender^®^ GmbH & Co. KG, Duisburg, Germany) at 190 °C and 260 °C for PP and PA6 nanocomposites, respectively, with 110 RPM for 10 min. Approximately 35 g per mixing was produced, containing the matrix polymer, nanoparticles, and a small spoonful of Irganox 1010:Irgafos 168 (2:1) antioxidants.

### 2.3. Characterization

Elemental analyses on carbon (C), hydrogen (H), and nitrogen (N) were performed on a Leeman Labs Inc. CE440 elemental analyzer (Teledyne Leeman Labs, Hudson, MA, USA) and Control Equipment Corporation 440 elemental analyzer (Control Equipment Co Inc., Marietta, GA, USA). Transmission electron microscopy (TEM) images were recorded digitally using a PhilipsTecnai 12 microscope (FEI, Santa Barbara, CA, USA) operating at 80 kV. Powder X-ray diffraction (XRD) patterns were recorded using a Siemens D-5000 wide-angle XRD spectrometer (LabX, Aubrey, TX, USA) with Cu Kα radiation, operating at 40 kV and 45 mA.

#### 2.3.1. Thermal Properties

Thermogravimetric analyses (TGA) were performed in an inert gas atmosphere (N_2_) using a SDT (TGA-DSC) Q600 thermal analyzer (TA instruments, New Castle, DE, USA). The samples were heated from 25 °C to 700 °C at a heating rate of 20 °C/min. Spherulites were observed by a microscope Leica DML (Leica microsystems, Buffalo Grove, IL, USA) with polarized light, provided with a heating station Linkam Scientific Instrument TMS 94 (Linkam Scientific Instrument, Tadworh, UK) controlled by a Linkam LTS 350 (Linkam Scientific Instrument, Tadworh, UK). Photographs were taken each minute with a Canon Power Shot A630 digital camera (Canon USA, Melville, NY, USA).

#### 2.3.2. Oxygen and Water Vapor Permeability

Samples for barrier properties were prepared by melt pressing the material at 190 °C and 260 °C for PP and PA6, respectively, and 50 bar in a heated hydraulic press for 5 min using a 0.2 mm stainless steel mold. The system was then cooled using a water cooling system to solidify the samples. Oxygen permeability testing was performed using the time-lag method in a permeability cell built in the polymer laboratory, where the design is described elsewhere [3,21]. The system was sealed hermetically and exposed to vacuum (10^−3^ bar) for 3 h before every test. Water vapor permeability was measured using the dry cup method [22]. Aluminum sample cups, equipped with rubber O-rings, were filled with 2 g of phosphorous penoxide and covered with a 3 cm diameter film of the polymer sample and secured above. The total mass of the cups was measured and then placed in a sealed chamber with 90% relative humidity and at 25 °C. The mass of the cups was measured every 24 h for 15 days. For both permeabilities, five measurements were performed for each nanocomposite.

#### 2.3.3. Mechanical Properties Testing

Samples were prepared by melt pressing the material at 190 °C and 260 °C for PP and PA6, respectively, and 50 bar in a heated hydraulic press for 5 min. Samples were then cooled using a water cooling system, solidifying the sample and releasing the pressure. Test samples were cut from these sheets using a stainless steel die in a hydraulic press, obtaining a 12 cm × 1 cm ×1 mm thickness testing sample, according to ASTM D638 [23]. Tensile tests under constant rate and creep conditions were measured using an HP D500 dynamometer (Dyne Systems, Jackson, WI, USA) with a strain rate of 50 mm/min at room temperature. Eight samples per nanocomposite were tested.

## 3. Results and Discussion

### 3.1. Characterization of Nanoparticles

Elemental analysis and specific surface area of the nanoparticles are shown in Table 1. The synthesized layered silica nanoparticles denoted “LSN” present the highest carbon content (63.6 wt %), confirming there are organic groups from the hexadecyltrimethoxysilane (HDTMOS) used in their synthesis, which are most likely responsible for their interlaminar distance [14]. Moreover, the ratio between carbon and hydrogen atoms in the hexadecyl group (C_16_) is ~17/3, similar to the ratio obtained by elemental analysis (~16/3). On the other hand, Cloisites C20A and C30B present 28.6% and 19.7% of organic material, respectively. The difference in the organic content is due to the surfactant as in the C20A there are two alkyl groups, whereas in the C30B there is just one. These results are similar to those shown elsewhere [2,11,13]. As a result, the C20A would be more compatible with the hydrophobic PP due to its higher organic content, while the C30B would be more compatible with the hydrophilic PA6. Regarding to the nitrogen content, both clays presented close values associated with the ammonium group present in both surfactants [1,2,11]. With respect to the specific surface, the C30B showed the highest specific area, meaning a better polymer–particle interaction [1,3].

**Figure 1:**
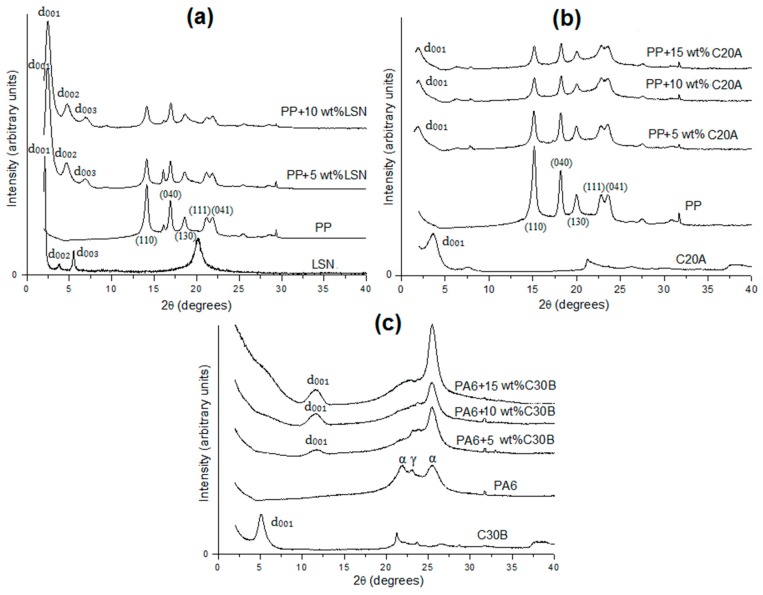
X-ray diffractograms of nanoparticles, polymer matrices, and nanocomposites: (**a**) PP/LSN; (**b**) PP/C20A; and (**c**) PA6/C30B.

The layered geometry of all of these nanoparticles was further confirmed by X-ray diffraction (XRD) as displayed in Figure 1. LSN (Figure 1a) presents an interlaminar distance (d_001_) of 4.8 nm associated with a diffraction peak at 1.9°, meaning that the inorganic layers are separated by a bilayer arrangement of hexadecyl groups (C_16_). In this case, 4.8 nm relates approximately to twice the length of the hexadecyl group [14]. The interlaminar distance of C20A (Figure 1b) and C30B (Figure 1c) are 2.5 nm and 1.8 nm, respectively, associated with diffraction peaks at 3.5° and 4.9°. These values are consistent with those delivered by Southern Clay Products Inc. The larger interlaminar distance in the C20A, with respect to the C30B, is due to the presence of two alkyl groups in its surfactant, as mentioned above.

**Figure 2:**
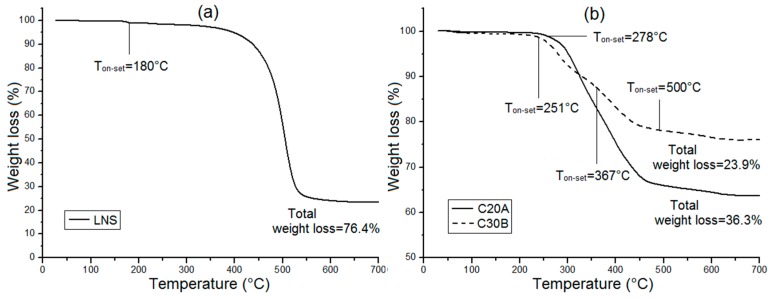
Thermogravimetric analysis (TGA) curves of the nanoparticles: (**a**) LSN; and (**b**) C20A and C30B.

Figure 2 shows the thermogravimetric analysis (TGA) curves of LSN (Figure 2a) and the clays (Figure 2b). In the case of the LSN, the degradation of the hexadecyl chains (C_16_) begins at ~180 °C with the greatest weight loss between 400 °C and 520 °C. The degradation of the C20A occurs in a single step, starting at 278 °C where the surfactant starts its decomposition [24]. In the C30B clays, its degradation occurs in three steps in the temperature range between 251 °C and 675 °C. The organic fraction decomposes to volatiles species in two steps between 251–376 °C and 376–500 °C, whereas the decomposition between 500 °C and 675 °C is due to the structural water loss [25]. As the onset of the LSN’s degradation is at 180 °C, it could be difficult to incorporate them with PA6 by melt-mixing (260 °C) due to the organic surfactant starting to degrade [26].

### 3.2. Nanocomposites

#### 3.2.1. Transmission Electron Microscopy (TEM)

**Figure 3:**
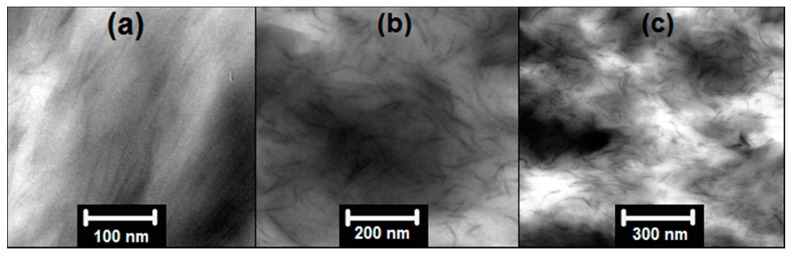
TEM images of nanocomposites with 10 wt % of nanoparticles: (**a**) PP/LSN; (**b**) PP/C20A; and (**c**) PA6/C30B.

Figure 3 displays some representative TEM images from the nanocomposites with 10 wt % of nanoparticles. Since the alkyl chains from LSN start their degradation at ~180 °C (Figure 2a) their melt mixing with PA6 at 260 °C was not possible. Moreover, the repulsive interaction between the apolar LSN and the hydrophilic PA6 matrix makes them difficult to mix. Figure 3 also shows that all of the nanocomposites present agglomerated nanoparticles (black areas in TEM images) with some degree of individual particles. In particular, PP nanocomposites (Figure 3a,b) with LSN and C20A nanoparticles showed a regular dispersion in the PP matrix due to the hydrophobic groups in these nanoparticles, increasing the affinity polymer–particle with a better dispersion of the C20A. In the case of C30B in PA6, Figure 3c illustrates both intercalated tactoids and partially-exfoliated platelets. This is because of the low organic content in the C30B, which make it more compatible with the hydrophilic PA6 matrix. Based on these TEM images, the length of the nanoparticles is estimated to be between 100 nm and 200 nm.

The XRD of the nanocomposites with different loadings of nanoparticles is displayed in Figure 1. In the PP/C20A diaffractogram (Figure 1b), the interlaminar distance of 2.5 nm associated with the diffraction peak at 3.5° of the C20A clays increases up to a value of 3.6 nm (2.4°) at 15 wt %, indicating an intercalation by PP [6,13,27]. This confirms the better dispersion of these nanoparticles compared to the PP/LSN nanocomposites, as showed in Figure 3. In the case of PP/LSN (Figure 1a) and PA6/C30B (Figure 1c) nanocomposites, the XRD indicated a slight decrease in the interlaminar distances, associated with higher angles. The PP/LSN nanocomposites reached values of 4.1 nm associated with a peak at 2.1°, meanwhile the interlaminar distance in the PA6/C30B nanocomposites decreased up to ~0.8 nm associate with a peak at 11.2°. An explanation could be that some of the organic molecules were expelled or degraded during the mixing process at 190 °C for PP and 260 °C for PA6 [26]. This is consistent with the onset of the degradation temperature observed in Figure 2, of ~180 °C and ~251 °C for LSN and C30B, respectively. This behavior has previously been observed in polycarbonate/MMT nanocomposites where a decrease from 2.5 nm to 1.3 nm of the interlaminar distance has been reported, caused by thermal degradation of the organic surfactant in the melt-mixing process at 250 °C [26].

Regarding the crystal phase of the polymer in the nanocomposites, XRD showed that the α-crystalline form of pure PP (Figure 1a,b) is not affected since in all cases, the monoclinic α reflections of PP can be found at 2θ angles of 14.2° (110 crystalline plane), 17.2° (040), 18.8° (130), 21.5° (111), and 21.9° (131 and 041) [27]. In the case of the PA6/C30B nanocomposites, it can be observed that the C30B clays had a profound effect on the crystallization due to the fact that they act as heterogeneous nucleating agents and accelerate the formation of α-phase crystals with the absence of γ-phase crystals [28]. This occurs because the nanoparticles accelerate the crystallization of the matrix and the α-phase crystals constituted the most thermodynamically stable phase in PA6 with a faster growth rate. Similar results have been found in PA6/MMT nanocomposites where the α-phase has been favored [28,29].

#### 3.2.2. Crystallization

**Figure 4:**
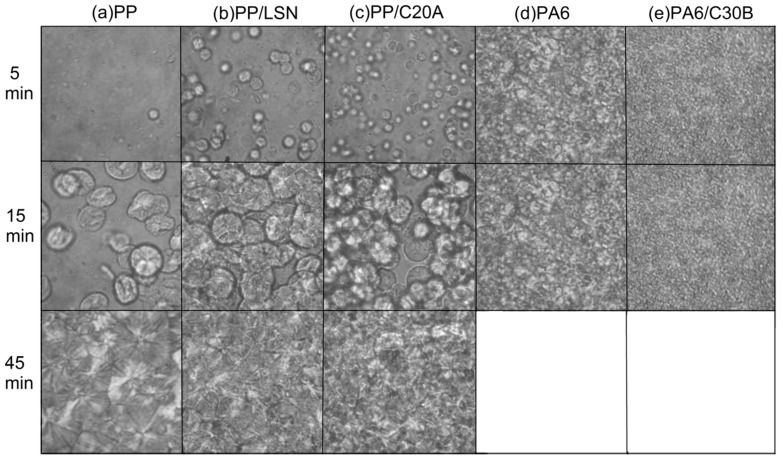
Optical micrographs showing the nucleating effect on the crystallization and the spherulite growth of the nanocomposites with 10 wt % at different times.

The results of Figure 4 evidence the influence of the nanoparticles in the polymer isothermal crystallization with 10 wt % of nanoparticles. As for pure PP (Figure 4a) and PA6 (Figure 4d), the spherulite sizes are larger and the spherulite densities are lower than their nanocomposites. With the addition of nanoparticles in these polymers, it is clear that their presence drastically increases the number of spherulites, resulting in a higher nucleation density. This is because incorporating a filler material usually increases the number of nucleation centers and, consequently, increased the number of spherulites [1,27]. In the PP nanocomposites (Figure 4b,c), the C20A clays had a better dispersion of spherulites than the LSN, as a result of the better dispersion of them in the PP, shown in Figure 3b. In addition, the presence of the LSN and C20A nanoparticles significantly increases the nuclei density of the PP, but has no discernible effect on its crystalline structure, as shown in Figure 1. In the case of PA6/C30B nanocomposites (Figure 4e), the addition of C30B also produced a smaller spherulite size and a larger number of them compared to that of the pure PA6 (Figure 4d). Similar results have been found in PA6/MMT nanocomposites [28]. In general, the presence of nanoparticles in the melting process can reduce both the work required to create a new surface and the nucleus size for crystal growth. This phenomenon is originated because the interface between the polymer crystal and the filler may be less hindered than the creation of the corresponding free polymer crystal surface, which increases the nucleation density of the spherulites [30]. These results show the strong effect of the nanoparticles on the nucleation density of PP and PA6, even if they are not well dispersed.

### 3.3. Permeability

#### 3.3.1. Oxygen Permeability

**Figure 5:**
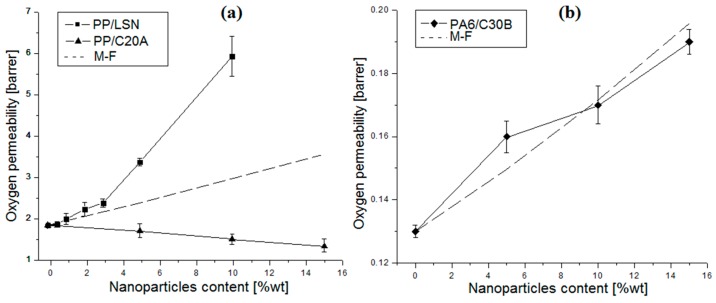
Oxygen permeability of nanocomposites: (**a**) PP/LSN and PP/C20A; and (**b**) PA6/C30B.

Results of oxygen permeability are presented in Figure 5. LSN (Figure 5a) strongly increased the oxygen permeability of PP and at 1 wt % the permeability increased by 10%. Meanwhile, at 10 wt % it was increased by a factor of ~3.5. It is also observed an increase of the oxygen permeability up to 46% at 15 wt % of C30B in PA6 (Figure 5b). However, a linear decrease in the oxygen permeability was observed in PP/C20A nanocomposites (Figure 5a), reaching decreases of 27.5% at 15 wt %.

For a better understanding, the modified Felske (M-F) model was used. This model predicts an increase of the permeability due to the void’s formation in the polymer–particle interface by a weak interaction. This model estimate the composite permeability by the following equations [31]:

(1) $$P_{c}=P_{m}\left[\frac{1+2\phi \left(\beta -\gamma \right)/\left(\beta +2\gamma \right)}{1-\psi \phi \left(\beta -\gamma \right)/\left(\beta +2\gamma \right)}\right]$$

(2) $$\psi =1+\left(\frac{1-\phi_{m}}{\phi_{m}^{2}}\right)\phi$$

(3) $$\beta =\left(2+\delta^{3}\right)\left(P_{p}/P_{m}\right)-2\left(1-\delta^{3}\right)\left(P_{i}/P_{m}\right)$$

(4) $$\gamma =2+\delta^{3}-\left(1-\delta^{3}\right)\left(P_{p}/P_{i}\right)$$

where $P_{m}$, $P_{p}$, and $P_{i}$ are the permeabilities of the polymer matrix (obtained experimentally), particles (considered impermeable), and voids (≈ 100 $P_{m}$ [31]), respectively; δ is the ratio of outer radius of interfacial shell to core radius [31] (1.45, “void size”); ϕ is the volume fraction of particles; and $\phi_{m}$ is the maximum amount of ϕ, which is considered 0.36 for layered nanoparticles [31].

It is noted that the M-F model, as displayed in Figure 5, significantly underestimated the experimental results of the PP/LSN nanocomposites, likely due to their geometry and high aspect ratio favoring void interconnection [1,20]. This can be associated with the “void size” that could be larger than assumed for this model (1.45). The model assumes an otherwise perfect dispersion of the filler material in the polymer matrix [31] where, in this case, it was not possible (Figure 3a). Additionally, the voids created by agglomerations could overlap with each other, forming preferential permeation channels, which could even go across the entire polymeric film, an effect known as percolation. In general, layered nanoparticles present geometrical percolation at low loadings, as reported elsewhere [32]. In the case of PP/C20A nanocomposites, they presented a diminution in the oxygen permeability that is probably due to the clay’s potential to act as impermeable barriers to gas molecules, forcing them to follow longer paths [7]. This behavior has previously been observed in similar PP/MMT nanocomposites with a good dispersion of clays [7,31,33]. It is worth noting that the permeability (*P*) typically proceeds by a solution-diffusion mechanism in which it can be calculated using *P* = *S*·*D* [1], where *S* and *D* denote the solubility and diffusivity of the permeating species, respectively. Therefore, an alternative to the decrease of the oxygen permeability in the PP/C20A nanocomposites, a diminution on the solubility of this apolar gas in the PP matrix as a result of the inorganic content in the C20A could also explain the experimental results [1,5]. In PA6/C30B nanocomposites, where the permeability increased, the M-F model predicted results similar to the experimental ones. Additionally, the organic content in the C30B increases the hydrophobicity of the PA6 matrix raising the solubility of apolar gases, such as O_2_.

#### 3.3.2. Water Vapor Permeability

**Figure 6:**
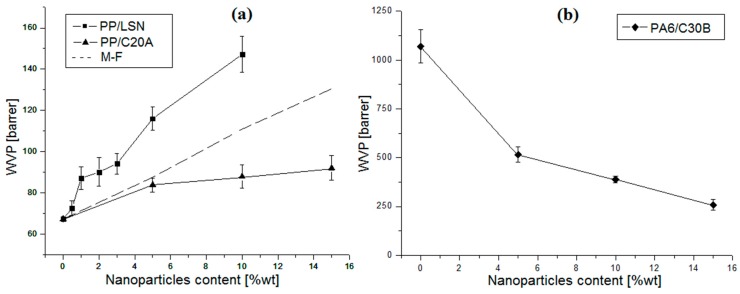
Water vapor permeability (WVP) of nanocomposites: (**a**) PP/LSN and PP/C20A; and (**b**) PA6/C30B.

Water vapor permeability (WVP) is shown in Figure 6. In the case of PP/LSN nanocomposites (Figure 6a) the WVP showed an increase of 12% and 40% at 1 wt % and 10 wt %, respectively. This is due to the void’s formation and channels between adjacent particles in systems with poor dispersion, as discussed above. This behavior has previously been observed in PP nanocomposites using different diameters of silica nanospheres, the product of the void’s formation, and channels between adjacent nanoparticles [3,4]. The WVP showed a similar trend obtained in the oxygen permeability (Figure 5a), although these increases are less pronounced, probably due to the presence of high organic content in the LSN, which have a better affinity to apolar gases, such as O_2_. In PP/C20A nanocomposites (Figure 6a) an increase was also observed, reaching 37% at 15 wt % of C20A which are contrary to what was obtained in the oxygen permeability (Figure 5a). In contrast, PA6/C30B nanocomposites showed a decrease in the WVP (Figure 6b) up to 76% at 15 wt % of C30B, which are also contrary with the oxygen permeability results (Figure 5b). In the case of the C20A, these clays have a high inorganic content (62.2% from Table 1) which may interact with polar gases such as H_2_O, increasing the solubility in the PP/C20A nanocomposites. In addition, the decrease in the WVP of the PA6/C30B nanocomposites is attributable to the organic content in the C30B (~20% from Table 1), suggesting that the water vapor (polar molecule) solubility in the PA6/C30B nanocomposites was decreased compared to the hydrophilic pure PA6. This can also explain the increase in the oxygen permeability in these nanocomposites (Figure 5b) where the PA6’s hydrophilicity was reduced, increasing the solubility of apolar gases, such as O_2_.

On the other hand, it is well known that in semicrystalline polymers, such as PP and PA6, the size, shape, and density of the spherulites have an influence on the permeation process. However, the impermeable units are layered crystals, not spherulites. The individual spherulite is, itself, a composite structure consisting of impermeable layered crystals arranged in a permeable interlamellar amorphous phase with lower density than the amorphous matrix. The basic assumption is that with smaller spherulite sizes, the permeability is minor [5,7,34]. Then, the decreases in the spherulite sizes in all of the nanocomposites found in Figure 4 expected a diminution in the permeability. However, although the oxygen permeability decrease in the PP/C20A and the WVP decreased in the PA6/C30B nanocomposites, there is no evidence supporting this approach since the permeability in those cases is led by the solubility, as recently discussed. Therefore, the nature of the nanoparticles has a significant effect on the permeability of the nanocomposites. In the PP nanocomposites, the synthetic LSN increased the permeability by diffusion because of the formation of preferential channels and voids in the nanocomposites, while the organically-modified C20A clay leads the permeability process by the solubility of the permeating gases. In the PA6/C30B nanocomposites, the organically-modified C30B clay also leads the permeability by the solubility of the permeating species.

### 3.4. Mechanical Properties

#### 3.4.1. Elastic Modulus

**Figure 7:**
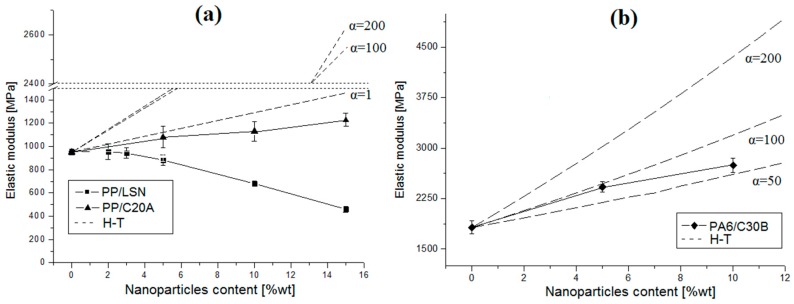
Elastic modulus of nanocomposites: (**a**) PP/LSN and PP/C20A; and (**b**) PA6/C30B.

The elastic modulus of the nanocomposites is illustrated in Figure 7. In the cases of PP/C20A (Figure 7a) and PA6/C30B (Figure 7b) nanocomposites, the Young’s modulus was linearly increased up to 28% and 50% at 15 wt % and 10 wt % of C20A and C30B, respectively. On the other hand, the Young’s modulus in PP/LSN nanocomposites decreased steadily up to 50% at 15 wt % of them.

For a better understanding, the results obtained were compared to the Halpin–Tsai (H–T) model, which estimates the composite yield strength using [9]:

(5) $$\frac{E_{C}}{E_{m}}=\frac{1+2\alpha \cdot \eta \cdot \phi }{1-\eta \cdot \phi }$$

(6) $$\eta =\frac{\left(E_{p}/E_{m}\right)-1}{\left(E_{p}/E_{m}\right)+2\alpha }$$

where ϕ is the particle volume fraction; α is its aspect ratio; $E_{p}$ and $E_{m}$ are the longitudinal stiffness for the particle and matrix (obtained experimentally), respectively; and $E_{C}$ is the composite longitudinal elastic modulus.

The layered nanoparticles have a high aspect ratio (α), in this case between 100 and 200 was found from Figure 3 and both were used in the H–T model. This model estimates the elastic modulus, assuming perfect adhesion between the particles and the polymer. In Figure 3 it was possible to observe some agglomerates of nanoparticles in the nanocomposites, decreasing the aspect ratio and, consequently, the increases in the elastic modulus of PP/C20A and PA6/C30B nanocomposites are lower than the estimate for the H–T model, even lower than the prediction for spherical geometry (α=1) in the case of the PP/C20A nanocomposites. The increase in the Young’s modulus with the clays cannot be explained by the adhesion mechanism since Figure 3 showed some agglomerates of these clays. Moreover, many studies have supported that the adhesion strength does not noticeably affect the elastic modulus when the particles have not been surface modified [2,35]. These increases can be due to the addition of rigid particles, such as clays, to polymer matrices which can easily improve the modulus since the rigidity of inorganic fillers is generally much higher than that of the organic polymers, a phenomenon known as the reinforcement effect [2,9,35]. In addition, the nanocomposite modulus consistently increases with the increase of the particle loading. The PA6/C30B nanocomposites have a greater enhancement in the elastic modulus than the PP/C20A nanocomposites due to the better dispersion of the C30B in the PA6 matrix. Similar results have been obtained in different nanocomposites with MMT [1,9,33,35]. In the case of PP/LSN nanocomposites, the decrease in the elastic modulus can be attributed to the formation of voids, as previously found in the permeability (Figure 5a and Figure 6a), agglomerations (Figure 3a), and nanocracks in the polymer matrix produced during the blending process, where the degradation of alkyl chains (C_16_) from the LSN begins at ~180 °C (Figure 2a), a temperature lower than the blending process temperature at 190 °C; then gases coming from its degradation can generate cracks. In the literature, a decrease in the elastic modulus has been reported by Altan, et al. [36] in similar nanocomposites (PP/TiO_2_) due to a poor polymer–particle interaction and agglomerate formation. Ash, et al. [37] have also reported a decrease in the elastic modulus in PMMA/alumina nanocomposites, a product of void formation and agglomerates of nanoparticles within the polymer matrix.

Regarding to the crystallinity of the nanocomposites, generally an increase in crystallinity or an increase in spherulite size increases the elastic modulus because large spherulites are envisaged to exhibit appreciably higher load-bearing ability [34]. In the PP/LSN and PP/C20A nanocomposites the nanoparticles had no influence in the crystallinity of PP, as shown in Figure 1. In addition, the spherulite size was significantly decreased. In the PA6/C30B nanocomposites, although the crystallinity was modified, the spherulite size was also decreased. The above observations suggest and confirm that it is the reinforcement effect that increases the elastic modulus in the PP/C20A and PA6/C30B nanocomposites since it has a positive effect on the elastic modulus, while the nucleating effect of the nanoparticles has a negative effect [34]. However, the mechanical behavior of nanocomposites is not straightforward and is not a simple function of spherulite size and crystallinity. In fact, it is a complex function of other factors, including lamellae thickness and the nature of the interface, among others [34,35,36,37,38].

#### 3.4.2. Yield Strength

**Figure 8:**
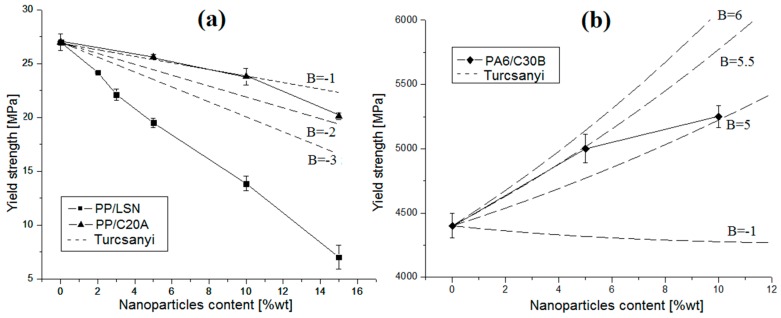
Yield strength of nanocomposites: (**a**) PP/LSN and PP/C20A; and (**b**) PA6/C30B.

Adding to the mechanical properties, Figure 8 shows the yield strength of the nanocomposites.

The results were compared with the Turcsanyi model, which estimates the composite yield strength using [38]:

(7) $$\sigma_{yc}=\sigma_{ym}\left(\frac{1-\phi }{1+2.5\phi }\right)\cdot \exp\left(B\phi \right)$$

where $\sigma_{yc}$ and $\sigma_{ym}$ are the yield stress of the composite and the matrix, respectively, ϕ the volume fraction of the filler in the composite, and B is related to the load carried by the dispersed component, i.e., it depends on the interaction.

At zero interaction (B=0) all of the load is carried by the polymer and the load-bearing cross-section decreases with the increase of filler content [38]. In Figure 8a, the strength of PP/LSN and PP/C20A nanocomposites decreases with the particle content. However, in the PA6/C30B nanocomposites (Figure 8b) the reverse is true, i.e., strength increases with the nanoparticle content. This contradiction is because, in addition to particle size and loading, the polymer–particle interfacial adhesion significantly affects the strength [35]. The decrease in the yield strength in the PP nanocomposites with LSN and C20A nanoparticles occurs because, in nanocomposites with poor/regular particle–polymer interaction (negatives values of B in the Turcsanyi model, as showed in Figure 8a) and agglomerations (previously mentioned), the applied stress cannot be effectively transmitted to the filler, and the energy is fully absorbed by the polymer matrix [1,13,16]. In the PA6/C30B nanocomposites, an increase of the yield strength was found where the Turcasanyi model can approximately estimate these results with a positive value of B, as a result of a better polymer–particle interaction, as seen in Figure 3. Moreover, the C30B clays showed the highest specific surface (9.7 m^2^/g from Table 1), which increase the probability of a better interaction between the C30B and the PA6’s chains. Therefore, a strong interfacial bonding between the nanoparticles and the polymer matrix is critical for an effective stress transfer leading to high nanocomposite strength. Vice-versa, a weak polymer–particle interface bonding will only give low nanocomposite strength.

### 3.5. Thermal Properties

Table 2 shows a summary of the thermogravimetric analysis (TGA) results for the prepared nanocomposites under inert conditions (N_2_). In all cases, a slight increase is seen on the onset of the degradation temperature (T_on-set_) and on the maximum degradation temperature (T_peak_).

According to the results of the Table 2, the nanoparticles do not influence in the thermal stability since the slight variations shown in the Ton-set and Tpeak are not of significance. Gilman, et al. found a diminution in the Ton-set and Tpeak in PA6/MMT nanocomposites due to agglomerations of MMT in the nanocomposites [13]. Most importantly, the study suggested that only exfoliated polymer nanocomposites exhibited improved thermal stability and agglomerated nanoparticles do not significantly affect the thermal stability of the polymer matrix. Thus, as a result of the agglomerated nanoparticles, as shown in Figure 3, the thermal stability in these nanocomposites does not present a significant variation. On the other hand, the final residue (at 700 °C) of the nanocomposites approximately matches the mass fraction of the incorporated nanoparticles. It indicates that the incorporation of the nanoparticles does not promote coke formation in the polymer degradation.

## 4. Conclusions

In this contribution, results of polypropylene (PP) and polyamide-6 (PA6) nanocomposites with layered silica nanoparticles (LSN) and organically-modified montmorillonites (C20A and C30B) provide relevant conclusions towards a better understanding of the final properties in polymer nanocomposites. The incorporation of filler material in the polymer matrices acted as centers of nucleation, increasing the density and the growth rate of spherulites in isothermal crystallization. In permeability, LSN in PP showed a significant increase in oxygen and water vapor permeabilities due to a percolation effect, as a result of agglomeration of these nanoparticles in the PP matrix, interconnecting voids (free volume) that can partially or fully traverse the membrane. In PP/C20A and PA6/C30B nanocomposites, the solubility has a more important role to play than the diffusivity, where the oxygen permeability increased in the PA6/C30Bnanocomposites due to these clays’ added hydrophobicity to the polar PA6 matrix. Meanwhile, the C20A in PP nanocomposites enhanced the water vapor permeability due to their inorganic content, adding hydrophilicity to the PP. Regarding the mechanical properties, C20A and C30B fillers increased the stiffness in PP and PA6, respectively, due to the load transferred from the reinforcement effect. In the case of LSN in PP, the formation of voids, agglomerations and the degradation of the alkyl chains of the LSN in the blending process can produce small cracks in the nanocomposites, which decrease the elastic modulus. These results expose the relevance between the type of layered nanoparticle and the polymer matrix, and also the importance of a good dispersion and interaction of them with the polymer, especially with respect to the barrier and mechanical properties.

## Figures and Tables

**Table 1:** Elemental analysis and specific surface of LSN, C20A, and C30B.

Nanoparticles	C	H	N	Inorganic content *	BET surface
	(wt %)	(wt %)	(wt %)	(wt %)	(m^2^/g)
LSN	63.6	12.0	-	23.6	8.1 ± 0.1
C20A	28.6	5.6	1.6	63.7	4.1 ± 0.4
C30B	19.7	3.7	1.7	76.1	9.7 ± 0.1

* Measured by TGA based on the remaining mass after heating to 700 °C in inert conditions (N_2_).

**Table 2:** The effect of the nanoparticle charges on the onset temperature and degradation peak temperature in the nanocomposites (20 °C/min, N_2_ atmosphere).

Load (wt %)	PP/LSN			PP/C20A			PA6/C30B		
	Ton-Set (°C)	Tpeak (°C)	Residue (%)	Ton-Set (°C)	Tpeak (°C)	Residue (%)	Ton-Set (°C)	Tpeak (°C)	Residue (%)
0	425.0	460.0	2.5	425.0	460.0	2.5	381.0	457.3	3.2
5	426.7	463.5	6.9	427.1	461.4	6.9	383.1	459.4	6.9
10	431.4	465.5	10.5	429.2	462.7	12.0	384.4	461.2	12.3
